# Pre-procedural high serum visfatin and tumor necrosis factor-α might predict recurrent atrial fibrillation after catheter ablation

**DOI:** 10.1186/s43044-023-00383-0

**Published:** 2023-07-19

**Authors:** Amr AlKassas, Mohamed Fouda, Gaetano Fassini, Mohamed Sanhoury

**Affiliations:** 1 Cardiology Department, Faculty of Medicine, Tanta University, Tanta, Egypt; 2 Clinical Pathology Department, Faculty of Medicine, Tanta University, Tanta, Egypt; 3 Cardiac Arrhythmia Research Centre, Department of Cardiovascular Sciences, Centro Cardiologico Monzino, University of Milan, Milan, Italy; 4 Cardiology Department, Faculty of Medicine, Alexandria University, Alexandria, Egypt

**Keywords:** Catheter ablation, Recurrent atrial fibrillation, Obesity, Inflammation, Adipocytokines

## Abstract

**Background:**

Many patients would require repeated ablation procedures owing to recurrent atrial fibrillation with its associated symptoms. Identifying those who are at risk of recurrent AF could assist us to develop preventive strategies and to properly select those who will benefit more from catheter ablation. Our aim is to study the role of preprocedural serum level of certain biomarkers in the prediction of AF recurrence after catheter ablation.

**Results:**

The present study included 117 patients: 26 patients with persistent and 91 patients with paroxysmal AF. Blood samples for estimation of serum levels of studied cytokines were obtained prior to the procedure. Pulmonary vein isolation was performed in all patients through point-by point radiofrequency ablation guided by 3D electroanatomical mapping system. Patients were followed for 12 months for AF recurrence. Forty-one (35%) patients developed AF recurrence. Those patients were significantly older, had significantly higher BMI, lower ejection fraction, and wider maximal left atrial diameter (LAD). Serum hs-CRP, IL-6, TNF-α, visfatin, and adiponectin levels were significantly higher compared to those who did not develop AF recurrence. Correlation analysis showed positive correlations between the incidence of RAF and patients’ age, BMI, and maximum LAD and elevated cytokine levels and maximal LAD showed significant correlations with the type of AF and elevated serum TNF-α, visfatin, and adiponectin. Statistical analyses defined elevated serum levels of TNF-α, visfatin, and adiponectin as positive predictors for RAF, and automatic linear modeling analysis showed that elevated serum visfatin, TNF-α, and adiponectin can predict RAF by accuracy rates of 50%, 34%, and 16%, respectively.

**Conclusions:**

RAF is most probably an outcome of the interplay between patients' clinical data, obesity, and inflammation. Pre-procedural estimation of serum levels of visfatin and TNF-α might determine patients with probability for RAF.

## Background

Atrial fibrillation (AF) is the most common clinical rhythm disorder [1]. AF is becoming increasingly ubiquitous and is associated with increased morbidity and mortality with advancing age [2] and reduces the quality of life because of associated symptoms, for instance, exercise intolerance, fatigue, and palpitation [3]. Thus, AF increases the threat to human health in addition to substantial medical and social costs.

Rhythm control therapy may slow down or even reverse progressive atrial remodeling [4]. Antiarrhythmic drug (AAD) therapy maintains sinus rhythm [5], but this approach has a failure rate added to side effects [2]. Catheter ablation (CA) procedures to treat atrial fibrillation in selected patients proved to maintain sinus rhythm and improve symptoms. Symptom improvement has been highlighted in the recent CABANA (Catheter ABlation vs. ANtiarrhythmic Drug Therapy for Atrial Fibrillation) RCT. CABANA revealed that catheter ablation as a strategy to treat AF failed to reduce the primary composite endpoints of death, stroke, serious bleeding, or cardiac arrest compared with a strategy of medical therapy alone [6].

However, the variability in CA techniques and energy sources may impact efficiency, safety, and efficacy; and the ideal strategy is still a matter of debate [7].

Pulmonary vein isolation (PVI) procedure is well-tolerated, effective, and promising for patients with paroxysmal and persistent AF with good acute procedural success, short procedure times, and acceptable safety [8]. CA is superior to AAD therapy for its significant reduction in recurrence rate of atrial arrhythmia in naive patients with paroxysmal AF and being associated with a similar rate of serious adverse events compared with AAD therapy [5].

In the case of persistent atrial fibrillation, PVI alone may result in unsatisfactory recurrence rates, so the left atrial posterior wall is a potential target for ablation in this population of patients [9]. Radiofrequency ablation creates irreversible myocardial injury through resistive heating, isolating the pulmonary veins or creating lines of functional block in the atria [10].

It is estimated that about 50–70% of patients undergoing atrial fibrillation ablation could maintain sinus rhythm at 1-year post-procedure. Many patients would require repeated ablation procedures owing to recurrent atrial fibrillation with its associated symptoms [11]. Thus, identifying those who are at risk of recurrent AF could assist us to develop preventive strategies and to properly select those who will benefit more from catheter ablation [12].

Regarding prediction of AF recurrence post RFA, many biomarkers have shown promising predictive role, but so far they are not routinely used. Visfatin is one of the previously described adipocytokines produced by visceral adipose tissue, and its concentration correlates with visceral obesity. It has a well established role in inflammatory response and fibrosis.

Multiple variables were previously evaluated and documented as predictors for RAF after CA of AF [13–16]; however, the incidence rate as previously mentioned is still high. These data illustrate the need for other factors to stratify AF patients according to the risk of recurrence. Thus, the current study hypothesized that estimation of serum levels of certain biomarkers might help to stratify AF patients regarding the possibility for getting recurrence after CA of AF.

## Methods

### Study cohort and covariables

All patients presenting to the outpatient clinics of the Cardiology Department, or referred from other centers for evaluation and management of AF in the period between October 2017 and August 2019 were eligible for evaluation for inclusion and exclusion criteria.

### Design

Prospective interventional comparative study. The study protocol was approved by the Local Ethical Committee. Trial registration number (NCT05114772).

### Exclusion criteria

History of longstanding persistent or permanent AF, myocardial infarction, acute coronary syndrome (ACS), significant heart failure (NYHA III), dilated or hypertrophic cardiomyopathy, left ventricular ejection fraction (LVEF) less than 35%, heart failure with preserved ejection fraction, congenital pathologies, significant valvular heart disease (more than moderate mitral valve stenosis or patients with mechanical heart valves), pulmonary embolism, venous thrombosis, intracardiac thrombus or inability to take warfarin or other oral anticoagulants, hepatic or renal insufficiency, acute inflammatory states (sepsis, COPD in acute phase), cancer, and autoimmune pathologies.

The previous conditions could increase the inflammatory response.

### Clinical evaluation

#### Diagnosis and classification of AF

Diagnosis of AF depended on an electrocardiogram (ECG). Transthoracic echocardiography was performed to estimate the maximum left atrial diameter (LAD), and normal value may reach up to 4.0 and 3.8 cm for men and women, respectively, as previously suggested by Leung et al. [17]. Also, the left ventricular ejection fraction (LVEF) was measured in all patients. AF was classified according to the ESC guidelines for the management of atrial fibrillation as paroxysmal AF (AF that terminates spontaneously or with intervention within 7 days of onset), or persistent (AF that is continuously sustained beyond 7 days, including episodes terminated by cardioversion (drugs or electrical cardioversion) after ≥ 7 days) [18].

#### Diagnosis of associated medical disorders

Hypertension was diagnosed if systolic and diastolic blood pressures were ≥ 140/90 mmHg at baseline visit; hyperlipidemia was diagnosed if total cholesterol or triglyceride levels were > 240 or 200 mg/dl, respectively; diabetes was diagnosed if fasting and 2-h plasma glucose levels were 126 and 200 mg/dl during oral glucose tolerance test, or if HbA1C is > 6.5%.

### Inclusion criteria

Symptomatic paroxysmal or persistent AF that was refractory to medical treatment (rhythm control) in patients who were free of exclusion criteria and accepted to sign the written fully informed consent to undergo CA and to give pre-procedural blood samples.

### Catheter ablation procedure

#### Preparation

- All patients received full oral anticoagulation for at least 1 month before the procedure using either warfarin or NOACs. Preoperative CT assessment of the left atrial and pulmonary vein anatomy and transesophageal Echo were performed 1 day before the ablation procedure, as per institutional protocol.
- Under conscious sedation, 6-F deflectable decapolar catheter (St. Jude Medical, Inc.) was placed within the coronary sinus, and after double transseptal puncture was performed using BRK needle and Swartz SL0 sheath (St. Jude Medical, Saint Paul, MN, USA), a 20-pole circumferential PV mapping catheter (Lasso, Biosense-Webster, Inc.) was placed inside the pulmonary veins to record PV signals and real-time PV isolation assessment. Left atrial geometry, as well as voltage mapping, were created with manipulation of the ablation catheter inside the LA cavity, LA-PV junctions, and in the proximal part of PVs.

#### Procedure

An open-irrigated, 3.5-mm tip, contact-force sensing radiofrequency (RF) ablation catheter (ThermoCool SmartTouch, Biosense Webster Inc.) was used, and with a power-controlled mode, ablation lesions were created by delivering 20–35 W (depending on anterior or posterior location) for 20–40 s per lesion during irrigation at a rate of 17–30 ml/min. We do not use esophageal temperature probe. A wide area of PV antral isolation was targeted. All lesion markers within the EAM were created using automated lesion annotation (VisiTag, Biosense Webster, Inc.). Ablation was continued until complete circumferential antral isolation and closure of all possible gaps in the ablation line. Entrance and exit block were both confirmed in every isolated vein. Heparin was given throughout the procedure to maintain an activated clotting time (ACT) of around 300 s. With patient with documented atrial flutter, Cavo-tricuspid isthmus isolation was done at the end of the procedure with achieving complete bidirectional block.

### Laboratory investigations

#### Sampling

Venous blood samples (5 ml) were collected under complete aseptic conditions. Blood samples were collected in a plain tube, allowed to clot, centrifuged at 1500×*g* for 15 min, and the serum samples were collected in a clean dry Eppendorf tube to be stored at − 70 °C until assayed.

#### Laboratory investigations

Serum levels of visfatin, adiponectin, TNF-α, IL-6, and hs-CRP were measured using enzyme-linked immunosorbent assay (ELISA) kits according to the manufacturer's instructions and were read using a 96 well microplate ELISA reader (Dynatech. MR 7000).

1. Human visfatin level using ELISA kit (catalog no. ab264623, Abcam Inc., San Francisco, USA) by quantitative sandwich enzyme immunoassay technique [19].
2. Human serum adiponectin level was estimated using ELISA kit (catalog no. ab99968, Abcam Inc., San Francisco, USA) by quantitative sandwich enzyme immunoassay technique [20].
3. Human TNF-α was measured with the enzyme-linked immunoassay (ELISA) kit (catalog no. ab46087, Abcam Inc., San Francisco, USA) by quantitative sandwich enzyme immunoassay technique [21].
4. Human IL-6 was measured with the enzyme-linked immunoassay (ELISA) kit (catalog no. ab187013, Abcam Inc., San Francisco, USA) by quantitative sandwich enzyme immunoassay technique [22].
5. Human CRP was measured with the enzyme-linked immunoassay (ELISA) kit (catalog no. ab99995, Abcam Inc., San Francisco, USA) by quantitative sandwich enzyme immunoassay technique [23].

### Follow-up

All patients were asked to attend the outpatient clinics at 3, 6, and 12 months. At each visit, a full clinical examination was performed and a 12-lead electrocardiogram, 24-h Holter monitoring, and at 12 months 7-day Holter monitoring was performed.

### Study outcome

1. The primary outcome was the incidence of AF recurrence that was defined according to the 2017 Heart Rhythm Society expert consensus statement as any AF or atrial tachycardia episode lasting longer than 30 s [24].
2. The secondary outcome is the relation between the recurrence rate and estimated serum levels of studied cytokines

### Statistical analysis

Data are presented as mean, standard deviation (SD), numbers, and percentages. Parametric results were analyzed using a one-way ANOVA test, and nonparametric results were analyzed using the Chi-square test. Correlations were studied using Pearson's correlation analysis considering the incidence of RAF as an independent variate and other constitutional, clinical and laboratory data as dependent variates. The receiver operating characteristic curve was used for the analysis of predictors for RAF. Automatic Linear Modeling analysis for serum levels of estimated cytokines as predictors for RAF. Statistical analysis was conducted using IBM® SPSS® Statistics (Version 22, 2015; Armonk, USA) for Windows statistical package. *p* value < 0.05 was considered statistically significant.

## Results

During the study duration, 139 patients who had AF refractory to medical treatment were evaluated, but 14 patients were excluded for not fulfilling the inclusion criteria and 8 patients were missed before CA, and 117 patients were prepared for the CA procedure. Clinical evaluation defined 26 patients who had persistent AF and 91 patients who had paroxysmal AF. All patients underwent the procedure successfully and passed the immediate post-procedural period and went home uneventfully. During 12-m follow-up, 41 patients had recurrent AF (RAF) with a recurrence rate of 35%; 29 patients (31.9%) had pre-procedural paroxysmal, and 12 patients (46.2%) had pre-procedural persistent AF with non-significantly (*p* = 0.178) higher incidence of RAF in patients who had persistent than paroxysmal AF. Median duration till recurrence of AF was 9 [IQR: 9–12] months; two patients (4.9%) had RAF 3-m after CA, 7 patients (17.1%) had RAF 6-m after CA, 13 patients (31.7%) had RAF after 9 months and 19 patients (46.3%) had RAF 12-m after CA.

Patients who had RAF were significantly older (*p* = 0.014), had significantly higher BMI (*p* = 0.0223), lower LVEF (*p* = 0.0318), and wider maximal LAD (*p* = 0.001), while other demographic and clinical data showed non-significant (*p* > 0.05) differences between patients who developed RAF or not (Table 1).

**Table 1:** Demographic and clinical data of studied AF patients categorized according to the outcome of CA

Variables	RAF (*n* = 41)	No RAF (*n* = 76)	Significance of difference
Type of AF			
Persistent (*n* = 26)	12 (46.2%)	14 (63.8%)	0.178
Paroxysmal (*n* = 91)	29 (31.9%)	62 (68.1%)	
Age (years)	68.3 ± 7.2	63.8 ± 10.1	0.014
Sex			
Males	33 (80.5%)	57 (75%)	0.501
Females	8 (19.5%)	19 (25%)	
Body mass index (kg/m^2^)	31.8 ± 3.1	30.3 ± 3.3	0.0223
Associated medical disorders			
Number			
No	7 (17%)	16 (21.1%)	0.759
One	25 (61%)	47 (61.8%)	
> 1	9 (22%)	13 (17.1%)	
Type			
Diabetes mellitus	15 (36.6%)	30 (39.5%)	0.887
Hypertension	18 (43.9%)	26 (34.2%)	
Heart failure	7 (17%)	13 (17.1%)	
Neurologic	3 (7.3%)	4 (5.3%)	
Left ventricular ejection fraction (%)	52.7 ± 4.8	54.9 ± 5.6	0.0318
Maximal Left atrial diameter (mm)	43 ± 3.9	40 ± 4.9	0.001
Systolic blood pressure (mmHg)	133.7 ± 10.7	130.1 ± 13.2	0.132
Diastolic blood pressure (mmHg)	86.4 ± 7	85 ± 7.3	0.314

Data are presented as mean, standard deviation, numbers, and percentages; RAF: Recurrent atrial fibrillation; *p* < 0.05 indicates a significant difference; *p* > 0.05 indicates a non-significant difference

Estimated blood glucose levels and HbA1c and lipid profile showed non-significant (*p* > 0.05) differences between patients who developed RAF or not. On the contrary, patients who developed RAF had significantly higher serum hs-CRP (*p* = 0.0016), IL-6 (*p* = 0.0216), TNF-α (*p* = 0.0002), visfatin (*p* = 0.0003) and adiponectin (*p* = 0.0019) levels in comparison with patients who completed their 12-m follow-up free of RAF (Table 2).

**Table 2:** Results of estimated Laboratory parameters in blood samples obtained at the time of admission of studied AF patients categorized according to the outcome of CA

Parameters	RAF (*n* = 41)	No RAF (*n* = 76)	Significance of difference (*p*)
Fasting blood glucose (mg/dl)	118.1 ± 13.3	117.4 ± 13.7	0.786
2-postprandial blood glucose (mg/dl)	173.6 ± 32.7	170.6 ± 35.5	0.651
Blood glycosylated hemoglobin A1c (%)	6.15 ± 0.63	6.3 ± 0.59	0.219
Total blood cholesterol (mg/dl)	235.5 ± 31	225.9 ± 31.5	0.116
Triglyceride (mg/dl)	173.2 ± 29.1	164.2 ± 27.3	0.079
High-sensitivity C-reactive protein (ng/ml)	6.8 ± 0.56	6.4 ± 0.7	0.0016
Interleukin-6 (pg/ml)	202.4 ± 27.5	189 ± 30.8	0.0216
Tumor necrosis factor-α (pg/ml)	2.01 ± 0.74	1.56 ± 0.52	0.0002
Visfatin (ng/ml)	7.54 ± 1.1	6.93 ± 0.78	0.0003
Adiponectin (µg/ml)	10 ± 3.79	8.15 ± 2.48	0.0019

Data are presented as mean; standard deviation; RAF: Recurrent atrial fibrillation; *p* < 0.05 indicates a significant difference; *p* > 0.05 indicates a non-significant difference

Correlation analysis showed positive significant correlations between the incidence of RAF and patients' BMI and maximum LAD and elevated serum levels of TNF-α, visfatin and adiponectin, patients' age, elevated serum levels of hs-CRP, triglycerides, and IL-6 and LVEF in decreasing order of significance, while other variable showed non-significant correlation. Considering maximal LAD as the objective predictor for RAF, it showed significant correlations with elevated serum TNF-α, visfatin, type of AF, and elevated serum adiponectin, in decreasing order of significance (Table 3).

**Table 3:** Pearson's correlation analysis of demographic, clinical, and laboratory variables and incidence of RAF

Variables	AF recurrence	
	“*r*”	*p*
Type of AF	0.124	0.181
Age	0.311	0.001
Male gender	0.062	0.506
BMI (kg/m^2^)	0.342	< 0.001
Maximal LAD (mm)	0.227	0.014
LVEF (%)	− 0.199	0.032
SBP (mmHg)	0.141	0.132
HbA1c (%)	0.114	0.221
Serum triglyceride (mg/dl)	0.264	0.004
Serum CRP (ng/ml)	0.288	0.002
Serum IL-6 (pg/ml)	0.212	0.022
Serum TNF-α (pg/ml)	0.337	< 0.001
Serum visfatin (ng/ml)	0.406	< 0.001
Serum adiponectin (µg/ml)	0.374	< 0.001

*p* value indicates the significance of “*r*”; *p* < 0.05 indicates significant difference; *p* > 0.05 indicates non-significant difference

*r* Pearson's correlation coefficient, *AF* atrial fibrillation, *LAD* maximal left atrial diameter, *LVEF* left ventricular ejection fraction, *SBP* systolic blood pressure, *HbA1c* glycosylated hemoglobin, *CRP* C-reactive protein, *IL-6* interleukin-6, *TNF-α* tumor necrosis factor-α

**Fig. 1:**
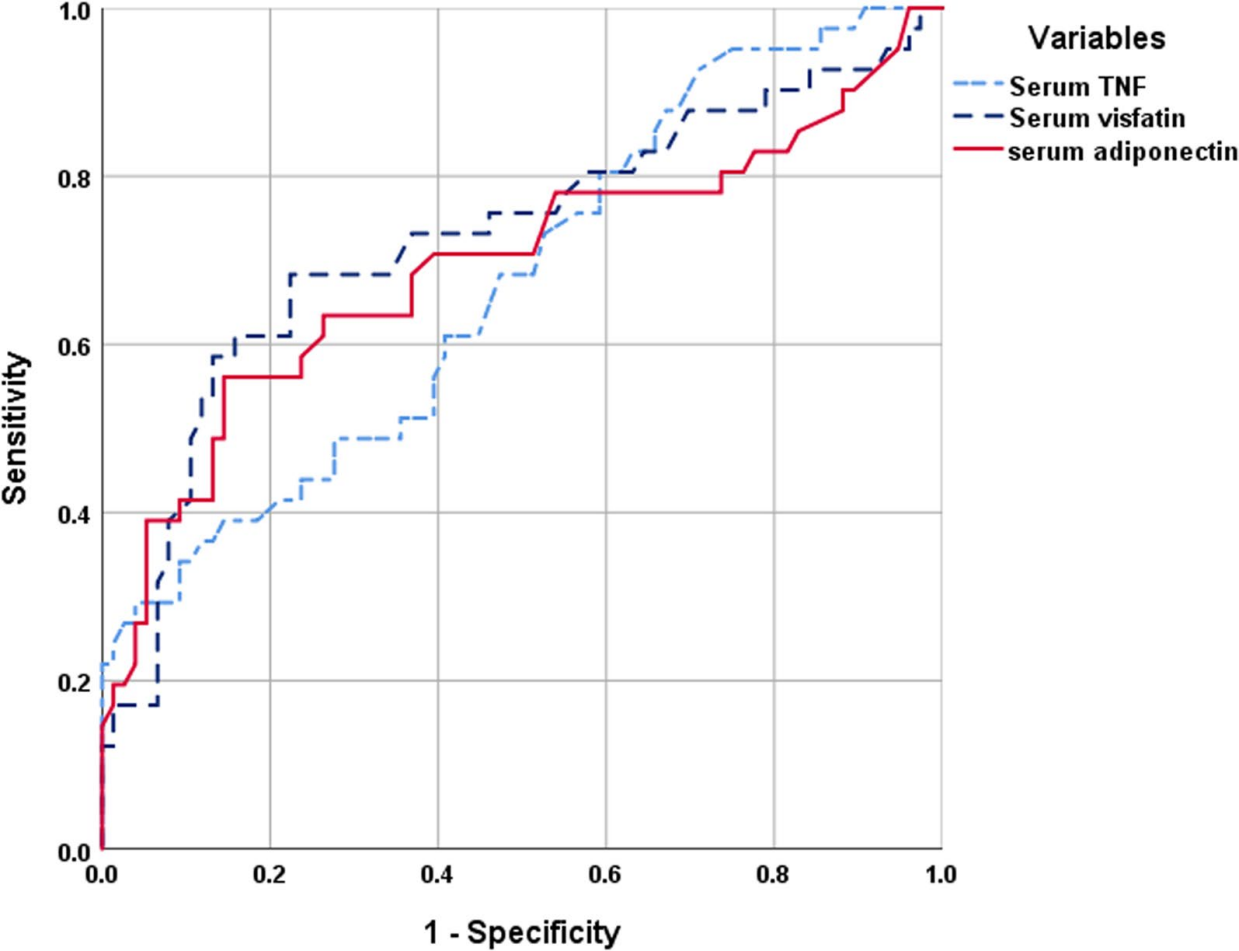
ROC curve analysis for positive predictive value (1-specificity) for upcoming RAF after CA as judged by the area under the curve

**Fig. 2:**
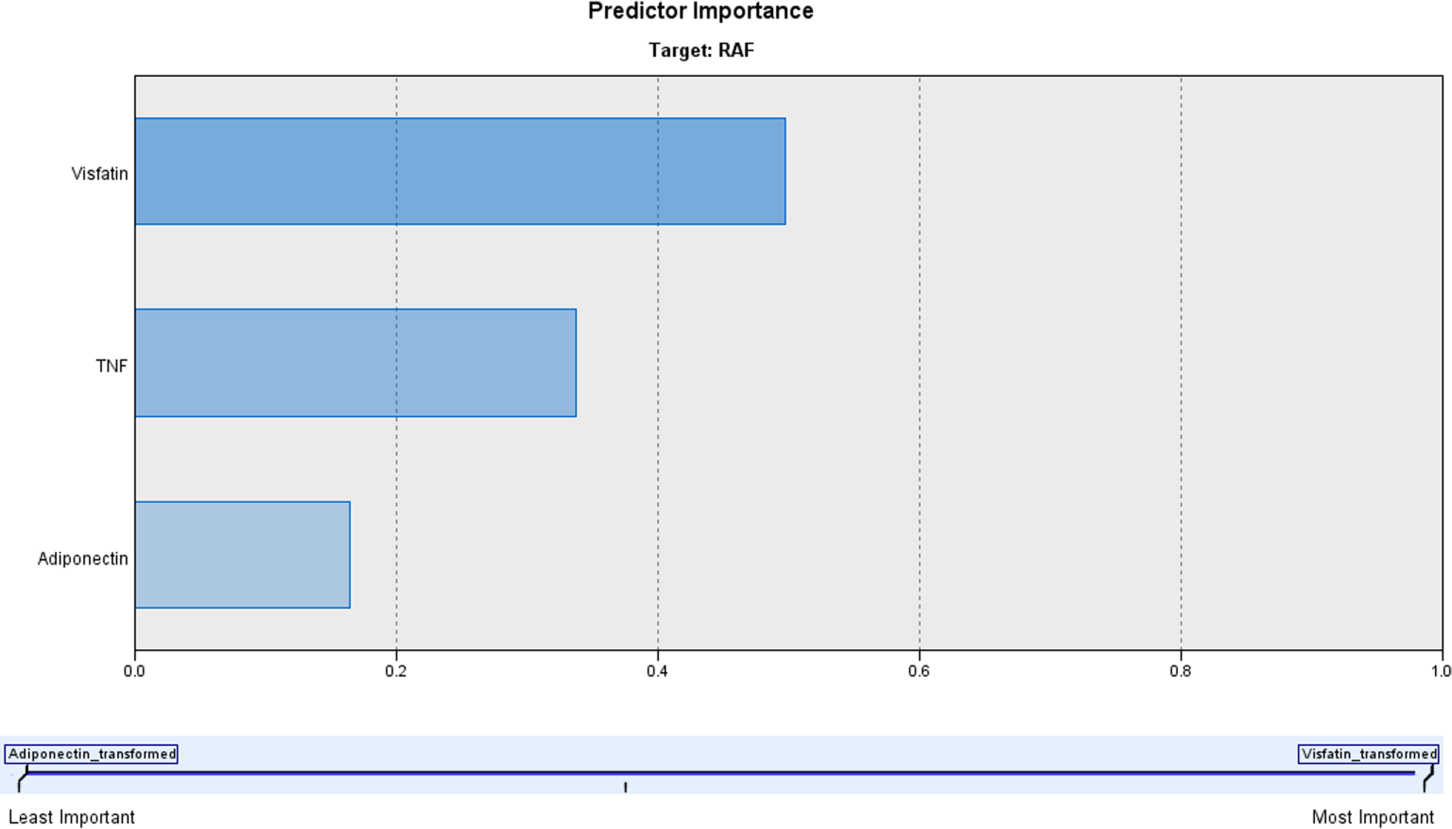
Automatic Linear Modeling analysis for the accuracy of predictors for upcoming RAF after CA

Regression analysis for variables correlated with the incidence of RAF defined elevated serum levels of TNF-α, visfatin, and adiponectin as the persistently significant positive predictor for upcoming RAF after CA. ROC curve analysis stratified these three predictors to define its positive predictive value for upcoming RAF after CA as follows: elevated serum visfatin, TNF-α, and adiponectin, in decreasing order of significance (Table 4, Fig. 1). Automatic Linear Modeling analysis assured the superiority of elevated serum visfatin as a predictor for upcoming RAF after CA with an accuracy rate of 50%, followed by elevated serum TNF-α and adiponectin with accuracy rates of 34% and 16%, respectively (Fig. 2).

**Table 4:** Regression and ROC curve analyses for predictors of upcoming RAF after CA

Variables	Regression analysis		ROC curve analysis		
	*β*	*p*	AUC (SE)	*p* value	95% CI
Serum TNF-α (pg/ml)	0.276	< 0.001	0.672 (0.52)	0.002	0.570–0.775
Serum visfatin (ng/ml)	0.345	< 0.001	0.728 (0.053)	< 0.0001	0.624–0.832
Serum adiponectin (µg/ml)	0.304	< 0.001	0.693 (0.056)	0.001	0.582–0.804

*p* value indicates the significance of β or AUC; *p* < 0.05 indicates a significant difference; *p* > 0.05 indicates a non-significant difference

*β* Standardized coefficient, *ROC curve* receiver operating characteristic curve, *AUC* area under curve, *CI* confidence interval, *TNF-α* tumor necrosis factor-α

## Discussion

The published figures for the incidence of RAF were discrepant; earlier studies reported RAF rate ranging between 18.7% [25] and 23.6% [26], while the recent figures for the incidence of RAF were 43% [27], 46.6% [28] and 54.7% [29]. During the 12-m follow-up period, the current study reported a total RAF incidence of 35% with a non-significantly higher incidence among patients who had pre-procedural persistent AF.

Patients who developed RAF were significantly older and had significantly larger LAD and RAF incidence that was significantly related to both variables. Similarly, Mlodawska et al. [30] reported that LAD ≥ 4.5 cm was significantly associated with RAF at follow-up and Chang et al. [31] detected that persistent AF and LAD are independently predicted recurrent atrial arrhythmia after ablation.

Moreover, patients who developed RAF had pre-procedural significantly higher BMI and RAF incidence was significantly correlated with BMI. These findings indicated a possible role for obesity in induction and/or recurrence of AF and supported earlier studies that documented prediction of higher recurrence after CA for non-paroxysmal AF patients with metabolic syndrome [32], and that hypertension, obesity, and long-standing persistent AF were associated with very-late RAF [33]. Also, Mlodawska et al. [30] had reported that the greater the BMI at baseline, the higher the likelihood of progression from paroxysmal to permanent AF, and Truong et al. [34] detected significantly higher number of overweight patients among hypertensive patients with RAF.

By the assumption that obesity is an important constitutional factor for induction, progression, or recurrence of AF, the current results showed significantly higher serum levels of studied adipocytokines; visfatin and adiponectin, and TNF-α in patients of RAF than in NO RAF groups. Moreover, the levels of these adipocytokines and studied primary phase reactants; CRP and IL-6 were positively correlated with LAD, which had previously been documented as an independent predictor for RAF [30, 31].

These findings illustrate the interplay between obesity and inflammation for atrial arrhythmogenesis and go in hand with Kim et al. [35] who found patients in the highest quartile of plasma adiponectin were more likely to be older and have greater LA volume index and high circulating adiponectin is independently associated with RAF after CA and with Guo et al. [36] who revised 6 cohort studies and found higher baseline circulating adiponectin may be an independent risk factor for the development of new-onset AF during follow-up. Recently, Zhu et al. [37] showed that high adiponectin level was an independent predictor of AF in the overall participants especially in women who were younger than 65 years.

Multiple recent studies, also, detected significantly higher levels of TNF-α, IL-6, CRP pre- and post-ablation with increased hazard ratios of RAF across increasing quartiles of these inflammatory biomarkers [38–40]. Also, Platek et al. [32] found patients with RAF had significantly higher visfatin levels and Chang et al. [31] detected a relation between resistin, an adipocytokine, and serum TNF-α, LAD, and RAF.

In line with the supposed risk factors interplay for induction of RAF, Zhao et al. [40] reported that in patients with paroxysmal AF without coexistent cardiovascular disease, enlarged LA volume and left ventricular myocardium abnormalities as detected by cardiovascular magnetic resonance were associated with inflammatory biomarkers and biomarkers of myocardial stiffness.

Statistical analyses of the obtained results defined high serum levels of visfatin, TNF-α and adiponectin as the persistently significant predictors for RAF, but elevated serum visfatin can predict RAF with accuracy rate of 50%, while the accuracy rate for elevated serum TNF-α and adiponectin as predictors for RAF was 34% and 16%, respectively.

In line with these findings, Platek et al. [29] using multivariate logistic regression analysis showed that patients with elevated visfatin levels were almost 3-time more likely to experience RAF and recommended the use of visfatin as a marker for risk stratification in AF patients undergoing CA.

In support of the importance of estimation of serum visfatin for AF patients, Szymanska et al. [41] detected significantly higher plasma visfatin levels in AF patients who were diagnosed with obstructive sleep apnea and apnea severity was correlated with visfatin serum level and recommended considering visfatin as a predictor for AF-associated complications.

Also, Zheng et al. [42] reported high serum visfatin levels in acute myocardial infarction patients and found these levels were correlated with an earlier onset and higher incidence of major adverse cardiovascular events. Moreover, Parimelazhagan et al. [43] found plasma visfatin levels were elevated in hypertensive patients with hypertriglyceridemia and associated with pro-inflammatory cytokines.

In a trial to explore the relationship between obesity and/or inflammation and induction, progression and recurrence of AF, Zhang et al. [44] found activation of the integrated stress response (ISR) pathway in the left atrium plays a key role in AF induction through activation of ISR pathway-related cardiac fibrosis, inflammatory macrophage infiltration, autophagy, and expression of ion channel and Cx43. Mahajan et al. [45] found sustained obesity was associated with increased LA pressure, inflammation, atrial transforming growth factor β1 protein, atrial fibrosis, epicardial fat infiltration, electrophysiological abnormalities, and AF burden and also reported that all these structural and electrophysiological changes could be reversed with reversed atrial remodeling and a reduced propensity for AF with weight reduction.

## Conclusions

Recurrent AF post catheter ablation is still a frequent and troublesome condition, and this necessitates proper patients selection. RAF is most probably an outcome of the interplay between patients' clinical data, obesity, and inflammation. Pre-procedural estimation of serum levels of visfatin and TNF-α might discriminate patients with probability for RAF.

## Data Availability

The datasets used and/or analyzed during the current study are available from the corresponding author on reasonable request.

## Abbreviations

TNF-α: Tumor necrosis factor-α
IL-6: Interleukin-6
hs-CRP: High-sensitivity C-reactive protein
CA: Catheter ablation
RAF: Recurrent AF
ELISA: Enzyme-linked immunosorbent assay
LAD: Left atrial diameter
BMI: Body mass index
NYHA: New York Heart Association
LA: Left atrium
LA-PV junctions: Left atrial-pulmonary vein junctions
EAM: Electro-anatomical mapping
